# The Role of *Drosophila* CtIP in Homology-Directed Repair of DNA Double-Strand Breaks

**DOI:** 10.3390/genes12091430

**Published:** 2021-09-16

**Authors:** Ian Yannuzzi, Margaret A. Butler, Joel Fernandez, Jeannine R. LaRocque

**Affiliations:** 1 Biology Department, Georgetown College, Georgetown University, Washington, DC 20057, USA; iy46@georgetown.edu; 2 Georgetown University Medical Center, Department of Human Science, Georgetown University, Washington, DC 20057, USA; mb2123@georgetown.edu (M.A.B.); Joel.Fernandez@georgetown.edu (J.F.)

**Keywords:** CtIP, double-strand break repair, homologous recombination, non-homologous end-joining, single-strand annealing, *Drosophila*, end resection

## Abstract

DNA double-strand breaks (DSBs) are a particularly genotoxic type of DNA damage that can result in chromosomal aberrations. Thus, proper repair of DSBs is essential to maintaining genome integrity. DSBs can be repaired by non-homologous end joining (NHEJ), where ends are processed before joining through ligation. Alternatively, DSBs can be repaired through homology-directed repair, either by homologous recombination (HR) or single-strand annealing (SSA). Both types of homology-directed repair are initiated by DNA end resection. In cultured human cells, the protein CtIP has been shown to play a role in DNA end resection through its interactions with CDK, BRCA1, DNA2, and the MRN complex. To elucidate the role of CtIP in a multicellular context, CRISPR/Cas9 genome editing was used to create a *DmCtIP*^Δ^ allele in *Drosophila melanogaster*. Using the DSB repair reporter assay direct repeat of *white* (DR-*white*), a two-fold decrease in HR in *DmCtIP*^Δ/Δ^ mutants was observed when compared to heterozygous controls. However, analysis of HR gene conversion tracts (GCTs) suggests DmCtIP plays a minimal role in determining GCT length. To assess the function of DmCtIP on both short (~550 bp) and long (~3.6 kb) end resection, modified homology-directed SSA repair assays were implemented, resulting in a two-fold decrease in SSA repair in both short and extensive end resection requirements in the *DmCtIP*^Δ/Δ^ mutants compared to heterozygote controls. Through these analyses, we affirmed the importance of end resection on DSB repair pathway choice in multicellular systems, described the function of DmCtIP in short and extensive DNA end resection, and determined the impact of end resection on GCT length during HR.

## 1. Introduction

Maintaining genome integrity is vital to ensuring proper cellular functions and the successful propagation of genetic material. This integrity relies on the efficient and accurate repair of DNA damage. A DNA double-strand break (DSB) is a distinctly cytotoxic type of DNA damage, where both strands of the DNA double helix are broken. DSBs can result from endogenous or exogenous sources. Endogenous sources include by-products of cellular processes (e.g., reactive oxygen species, single-strand breaks converted to DSBs during replication) or programmed DSBs (e.g., during meiosis and V(D)J recombination); these endogenous sources of DSBs account for about 50 DSBs per cell division [1]. Exogenous sources include UV radiation, ionizing radiation, and chemical reagents. Without proper repair of DSBs, cell death, chromosomal rearrangements, or carcinogenesis can occur [2,3]. Thus, there are multiple pathways to maintain genome integrity: non-homologous end-joining (NHEJ), homologous recombination (HR), and single-strand annealing (SSA).

In NHEJ, the two DSB ends are recognized, processed, and ligated together—often resulting in the loss or addition of nucleotides at the break site. In contrast to NHEJ, homology-directed repair (HDR) requires homologous sequences to repair DSBs. HDR is initiated by 5′ to 3′ end resection. If the DSB has occurred between direct sequence repeats with complementary base pairing, this extensive end resection is followed by SSA, where the two strands are annealed together. SSA does not require strand invasion or repair synthesis [4], but often involves losing the genetic information between the direct repeats; thus, this repair pathway is also considered error-prone. 

In contrast, HR is considered error-free due to its use of an unbroken donor sequence (in the form of a sister chromatid or homologous chromosome) to guide repair. HR is initiated following 5′ to 3′ end resection, when the 3′ single-strand overhangs invade the donor template through their interaction with RAD51 (and DMC1 in meiotic cells), resulting in the initiation of repair synthesis and the formation of heteroduplex DNA (hDNA) [5]. After initiating repair synthesis, HR can follow two distinct models: double-strand break repair (DSBR) or synthesis-dependent strand annealing (SDSA). DSBR is utilized in meiotically dividing cells, where the resolution of the double Holliday junction can result in either a crossover or noncrossover product [6]. SDSA, where the newly synthesized strand dissociates from the homologous template and anneals to the other end, is preferred in mitotically dividing cells [7,8,9]. The remaining gap on the other strand is synthesized, and the nicks are ligated together. In both DSBR and SDSA, correction of mismatched base pairs in hDNA to that of the homologous donor sequence results in gene conversion tracts (GCTs). 

The choice between DSB repair pathways depends on the cell cycle, chromatin context (i.e., euchromatin or heterochromatin), and end resection [10,11,12,13]. Generally, HR is favored in the S/G2 phases of the cell cycle, when the sister chromatid is available as a donor template [14], although both HR and NHEJ coexist throughout the cell cycle [15]. In mammalian cells, the mechanism behind this cell-cycle-dependent regulation has been shown to involve a regulatory circuit with 53BP1-RIF1 and BRCA1-CtIP [12,15,16]. In the G1 phase, phosphorylated 53BP1 recruits RIF1 to the DSB break site, thus preventing end resection and promoting NHEJ. In the S/G2 phases, CDK phosphorylates CtIP, allowing CtIP to associate with BRCA1 and displace RIF1 and 53BP1 from the break site. This displacement facilitates the recruitment of the MRE11-RAD50-NBS1 (MRN) complex, which initiates end resection [17]. In human cell lines, it is suggested that phosphorylated CtIP is involved in both the initial and extensive end resection that commits repair to homology-directed repair [18,19].

While CtIP has been identified as an endonuclease, its role in end resection is also connected to promoting the function of other endonucleases [19]. In the initial end resection, phosphorylated CtIP activates the endonuclease activity of MRE11, allowing for the removal of end-bound proteins, such as Ku, which is required for NHEJ, as well as the processing of DNA secondary structures [20]. The kinetically faster and more extensive end resection is facilitated by either the EXO1 or DNA2 endonucleases in conjunction with the Bloom (BLM) or Werner (WRN) helicases [21]. Specifically, phosphorylated CtIP may be involved in the DNA2 pathway by promoting the unwinding of DNA by BLM and the motor activity of DNA2 [22]. The motor activity of DNA2 involves translocating from 5′ to 3′ on ssDNA to displace the ssDNA binding protein RPA, which is required before degradation of the 5′ strand [23]. While progress has been made in elucidating the role of CtIP in in vitro biochemical assays and mammalian cell lines, understanding CtIP in a multicellular, whole organism context remains elusive. 

In this study, we used *Drosophila melanogaster* to understand the impact of DmCtIP on DSB repair pathway choice, focusing on its role in end resection during HDR. Previous studies have demonstrated that homozygous deletion of *CtIP* orthologs in mammals results in embryonic lethality [24]. However, a cluster analysis comparing CtIP protein alignments among eukaryotes shows that the *Drosophila CtIP* ortholog is uniquely diverged [25], and it is nonessential in *Drosophila* (this study). Thus, we were able to elucidate the role of CtIP in a multicellular organism. Using the direct repeat of *white* (DR-*white*) and direct repeat of *white with mutations* (DR-*white.mu*) DSB repair assays, we determined the relative frequencies of noncrossover HR and GCT length after an I-SceI-induced DSB in *DmCtIP*^Δ/Δ^ mutants and heterozygote controls. To understand the importance of *DmCtIP* in extensive versus short end resection, we executed modified SSA DSB repair assays. Our results implicate a role for DmCtIP in HR and SSA but not in determining GCT length, furthering our understanding of end resection and homologous recombination in the multicellular context. 

## 2. Materials and Methods

### 2.1. Drosophila Stocks and Maintenance 

*Drosophila* were maintained on standard Nutri-fly Bloomington Formulation medium (Genesee Scientific, San Diego, CA, USA) at 25 °C with 12 h day/night cycle. DR-*white* and DR-*white.mu* transgenic stocks were previously described [26,27,28,29]. The P{*70I-SceI*} transgenic stocks contain an *I-SceI* meganuclease transgene expressed by a *Drosophila hsp70* promoter for heat shock induction [30,31]. The P{*w*I*w*} and P{*w*I*w*Δ*AvrII*} assay stocks were gifts from Jeff Sekelsky (UNC-Chapel Hill) [32,33].

### 2.2. Molecular Analyses

For genotyping and gene conversion tract analyses, genomic DNA was isolated from individual flies using 50 µL Squishing Buffer (10 mM Tris-Cl, 25 mM NaCl, and 1 mM EDTA) and Proteinase K (0.2 mg/mL). Samples were incubated at 37 °C for 30 min, followed by inactivation of Proteinase K at 95 °C for 2 min [34]. To genotype for *DmCtIP* alleles, primers *DmCtIP_*66f (forward, 5′-GGTCGGCTAACAAATACCAACC) and *DmCtIP_*1848a (reverse, 5′-GGTCCCAAAACCGAGTGTCT) were used to screen for *DmCtIP* deletions, with an expected PCR product of approximately 284 bp. Primers *DmCtIP_*1378f (forward, 5′-CCCCAAAAGTTGAGAGCGTC) and *DmCtIP_*1848a (reverse) were used to screen for the wild-type locus, as amplification of the expected 470 bp product would only occur in the presence of a wild-type *DmCtIP* sequence. PCR was completed with SapphireAmp Fast PCR Master Mix (Takara Bio) with the following cycling conditions: 94 °C, 3 min; [94 °C, 30 s; 66 °C, 30 s; 72 °C, 5 s] × 16; [94 °C, 30 s; 58 °C, 30 s; 72 °C, 5 s] × 20; 72 °C, 5 min and confirmed by gel electrophoresis (1% TAE agarose gel, 150Vh). 

### 2.3. Establishing DmCtIP Mutants

*DmCtIP* mutants were created using CRISPR/Cas9 with the pCFD4-U6:1_U3:1 plasmid (Addgene #49411) [35]. gRNA sequences were determined using flyCRISPR (https://flycrispr.org/) (accessed on 23 August 2018) to minimize off-target events. Tandem gRNAs that targeted the stop and start codon of the *DmCtIP* gene were cloned into the expression vector (forward, 5′-TATATAGGAAAGATATCCGGGTGAACTTCGTTGTAAAAAAGATGACGTGGTTTTAGAGCTAGAAATAGCAAG; reverse, 5′-ATTTTAACTTGCTATTTCTAGCTCTAAAACGGCGGAGTTGAACTGCGAGCGACGTTAAATTGAAAATAGGTC), creating the pCFD4_*DmCtIP*_gRNA vector. Sanger sequencing was used to screen for proper integration of gRNAs into pCFD4 (Genewiz, South Plainfield, NJ, USA). The purified pCFD4_*DmCtIP_*gRNA expression vector was microinjected into *Cas9*-expressing embryos (*y w*; *nos-Cas9 (II-attP40) y +* /*CyO*) by BestGene, Inc. (Chino Hills, CA, USA). Potential *DmCtIP*^Δ^ alleles were isolated by crossing 12 single male G0 progeny to *TM3/TM6B* virgins; G1 progeny were crossed again to *TM3/TM6B* virgins for a total of 96 G1 single male crosses. Deletion events were identified through PCR using *DmCtIP_*66f and *DmCtIP_*1848a. Positive deletion events were then sequenced (Genewiz) to analyze the specific deletion events. Sequences were analyzed using 4Peaks software (Nucleobytes, Aalsmeer, The Netherlands).

*DmCtIP^9C^* and *DmCtIP^9E^* alleles were isolated as independent events (i.e., different G1 males) but resulted in the same molecular deletion due to the precise nature of gRNA associated CRISPR/Cas9 cleavage. For all experiments, null mutants were *DmCtIP^9C^* and *DmCtIP^9E^* trans heterozygotes, herein referred to as *DmCtIP*^Δ/Δ^ mutants. 

### 2.4. Establishing Recombinant Stocks

Recombinant stocks were created through standard *Drosophila* genetics to integrate *Sb*, P{*70I-SceI*}, and *DmCtIP^9C^* on Chromosome *3*. P{*w*I*w*} or P{*w*I*w*Δ*AvrII*} and *DmCtIP^9E^* were also recombined together on Chromosome *3*. PCR was used to screen for recombinants with primers *DmCtIP_*66f, *DmCtIP_*1848a, I-SceI_1a (5′-CGCAGACCCTTAACCAGGTA), and I-SceI_1 (5′-CCAGCTGATCGAACTGAACA). The DR-*white* and DR-*white.mu* assays integrated on Chromosome *2* were then crossed into the *DmCtIP^9E^^/9E^* mutant background through standard *Drosophila* genetics. 

### 2.5. DR-white and DR-white.mu Assays

To induce DSBs, females homozygous for *DmCtIP^9E^* containing DR-*white* or DR-*white.mu* were crossed to males heterozygous for *DmCtIP^9C^* containing P{*70I-SceI*}. After 3 days, flies were removed, and 0–3-day-old embryos were then heat-shocked at 38 °C for 1 h. Single F1 males, containing both DR-*white* (or DR-*white.mu*) and P{*70I-SceI*}, that were heterozygous for either *DmCtIP^9E^* or *DmCtIP^9C^* (*DmCtIP*^Δ*/+*^), or *DmCtIP*^Δ/Δ^ mutants were crossed to five tester *y w* females in vials. For each experiment, F2 progeny from 14–28 individual F1 male germlines of each genotype were scored (~20–60 progeny/germline). Genotypes of F1 males were confirmed by isolating genomic DNA and performing PCR followed by gel electrophoresis for visualization (see Section 2.2). 

### 2.6. GCT Analysis

For molecular analyses of gene conversion tracts, one or two DR-*white.mu* HR events (*y^+^ w^+^)* of F2 progeny from each F1 male germline were analyzed. The number of events per F1 germline was limited to avoid frequency biases attributable to potential germline jackpot events [36]. Repair events were amplified after genomic DNA extraction with DR-white_1 (forward, 5′-GTGTGAAAAATCCCGGCA) or DR-*white_*1.3 (forward, 5′-GTTTTGGGTGGGTAAGCAGG) and DR-*white_*1a (reverse, 5′-AGACCCACGTAGTCCAGC) using SapphireAmp Fast PCR Master Mix (Takara Bio). PCR cycling conditions were the same as for genotyping. PCR products were directly sequenced (Genewiz) with primers DR-*white_*2a (5′-TGGCAACCATCGTTGTCTG), DR-*white_*5a (reverse, 5′-GGATCGAAATTGATGATC), and DR-*white_*1a to detect incorporations of any of the 28 silent polymorphisms from the *iwhite.mu* donor sequence. Sequences were analyzed using 4Peaks software (Nucleobytes, Aalsmeer, The Netherlands).

### 2.7. SSA Assays

The P{*w*I*w*} and P{*w*I*w*Δ*AvrII*} SSA assays were performed as previously described [32,33]. Briefly, females containing P{*w*I*w*} (or P{*w*I*w*Δ*AvrII*) that were heterozygous for *DmCtIP^9E^* (*DmCtIP*^Δ*/+*^) were crossed to males containing P{*70I-SceI*} that were heterozygous for *DmCtIP^9C^* (*DmCtIP*^Δ*/+*^). After 3 days, flies were removed, and 0–3-day-old embryos were then heat-shocked at 38 °C for 1 h. Single F1 males containing both P{*wIw*} (or P{*w*I*w*Δ*AvrII*) and P{*70I-SceI*} that were heterozygous for either *DmCtIP^9E^* or *DmCtIP^9C^* (*DmCtIP*^Δ*/+*^), or *DmCtIP*^Δ/Δ^ mutants were crossed to five tester *y w* females in vials. For each experiment, F2 progeny from 22–26 individual F1 male germlines of each genotype were scored (~20–100 progeny/germline). Genotypes of F1 males were confirmed by isolating genomic DNA and performing PCR followed by gel electrophoresis for visualization (see Section 2.2, Molecular Analyses).

## 3. Results

### 3.1. DmCtIP Facilitates Repair of DSBs through Homologous Recombination

**Figure 1:**
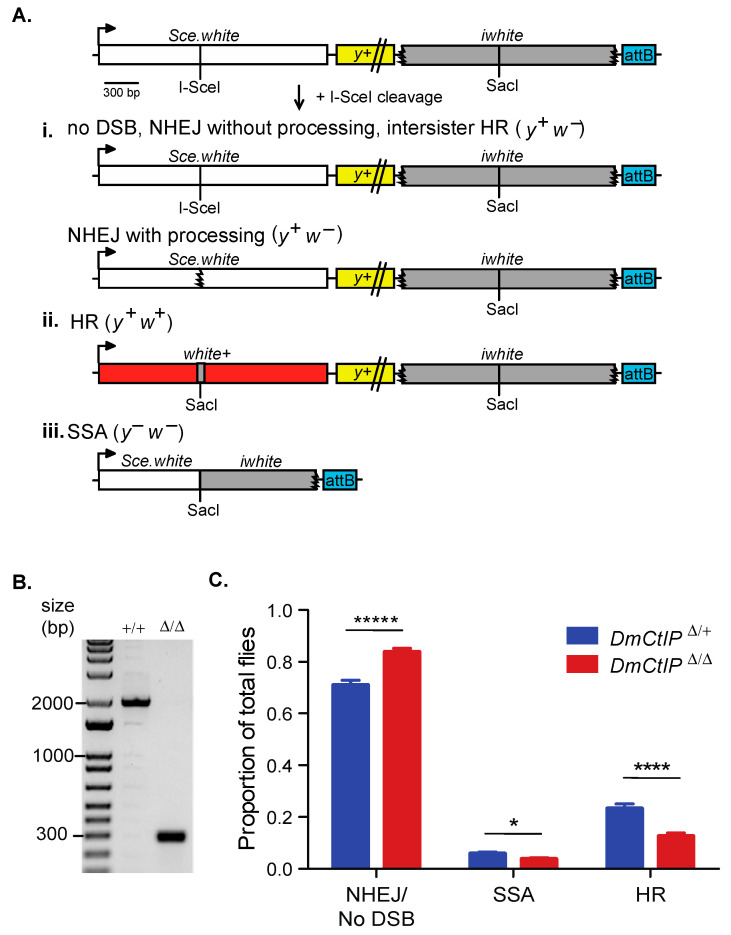
DR-*white* assay suggests defects in homologous recombination in *DmCtIP*^Δ/Δ^ mutants. (**A**) The direct repeat of *white* (DR-*white*) assay contains two non-functional *white* repeats: *Sce.white* (I-SceI recognition site and premature STOP codon) and *iwhite* (truncated donor sequence). DR-*white* is integrated into the genome at Chromosome *2* at a known attP landing site using the attB sequence (blue) and followed using the *yellow* (*y*^+^) transgene. Embryos and larvae containing DR-*white* and the heat-shock inducible *I-SceI* transgene are heat shocked, and a DSB is formed at the I-SceI site. Repair events are observed by crossing single males to *y w* tester females. The resulting progeny are representative of single double-strand break (DSB) repair events from the premeiotic male germline. Depending on the repair pathway, one of three phenotypes will result. (i) White-eyed progeny (*y^+^ w^−^*) are indicative of no DSB, intersister homologous recombination (HR), or non-homologous end joining (NHEJ). NHEJ with processing can be determined through molecular analysis. (ii) Red-eyed progeny (*y^+^ w^+^*) indicate repair by intrachromosomal HR with the *iwhite* sequence as the donor, restoring the function of the *white* gene. (iii) Yellow-bodied, white-eyed progeny (*y^−^ w*^−^) indicate repair by single-strand annealing (SSA), a mitotic crossover event (indistinguishable from SSA), or an abnormal repair event that inhibits *y*^+^ expression, such as a deletion into the *y*^+^ transgene. (**B**) PCR amplification across the CRISPR/Cas9-mediated deletion of *DmCtIP*. Primers produce 1934 bp product in wildtype (+/+) and 283 bp product in *DmCtIP*^Δ/Δ^ mutants (Δ/Δ) (**C**) I-SceI-induced DSB repair events in a *D. melanogaster* C-terminal Binding Protein 1 Interacting Protein (*DmCtIP*^Δ/Δ^) mutant background (red; *n* = 28 germlines, 1520 total flies scored) compared to *DmCtIP*^Δ*/+*^ heterozygote controls (blue; *n* = 28 germlines, 1289 total flies scored). Results shown are averages ± standard error of the mean (S.E.M.) of individual male germline events. * *p* < 0.05, **** *p* < 0.0001, and ***** *p <* 0.00001 by unpaired Student’s *t*-test.

The DR-*white* assay allows for assessing the usage of intrachromosomal HR, SSA, or NHEJ/no DSB/intersister HR in the repair of a site-specific DSB as described previously (Figure 1A) [26]. Briefly, the DR-*white* assay contains two non-functional repeats of the *white* gene. The upstream *white* sequence, *Sce.white*, is non-functional due to the insertion of 23 bps containing the I-SceI recognition site and resulting in a premature stop codon. The downstream *white* sequence, *iwhite*, lacks the 5′ UTR, ATG start site, 30 amino acids on the carboxy-terminal end as well as the 3′UTR.

Expression of I-SceI results in cleavage at the I-SceI recognition site on *Sce.white,* inducing a DSB that is then repaired [37]. Repair events in the premeiotic germline cells can be captured by crossing out to *y w* tester females. Progeny of this cross represent single repair events that can be distinguished phenotypically. If there is no DSB formation, repair by NHEJ, or repair by intersister HR, the progeny have brown bodies and white eyes (*y*^+^ *w*^−^; Figure 1A(i). NHEJ with processing and microhomology-mediated end joining (MMEJ) can be detected through amplifying and sequencing across the I-SceI site. The loss of the I-SceI recognition site indicates repair by NHEJ with processing (indels), and can include the annealing of sequences with microhomologies between 8 and 20 nucleotides long, which suggests MMEJ [38,39]. If accurate repair by intrachromosomal noncrossover HR occurs, the I-SceI recognition sequence is converted to the wild-type SacI sequence from *iwhite* and *w^+^* expression is restored—resulting in brown-bodied, red-eyed (*y^+^ w^+^*) progeny (Figure 1A(ii). Lastly, if an SSA event occurs in the DR-*white* assay, the two *white* sequence repeats are annealed after extensive end resection (~7.4 kb), causing the loss of the intervening *y^+^* gene and resulting in yellow-bodied, white-eyed (*y*^−^ *w*^−^) progeny (Figure 1A(iii). Loss of the *y*^+^ transgene could also occur through an aberrant repair event (e.g., deletion) or a mitotic crossover event, although these are suppressed in wild-type cells [27].

To understand the role of *D. melanogaster* CtIP in DSB repair pathway choice, we tested complete knockout *DmCtIP* mutants (*DmCtIP*^Δ/Δ^) with the DR-*white* assay. The *DmCtIP*^Δ/Δ^ mutants contain a CRISPR/Cas9-mediated deletion of 1651 bp in the *DmCtIP* coding region (Figure 1B). The deletion includes all sequences except the ATG start site of the *DmCtIP isoform B* and deletion of amino acids 2 to 455 (out of 483) of the DmCtIP isoform A, with the last remaining amino acids out of reading frame. 

We found a ~50% decrease of noncrossover HR events, from 23.2 ± 1.8% in the *DmCtIP*^Δ*/+*^ heterozygote controls to 12.5 ± 1.3% in the *DmCtIP*^Δ/Δ^ mutants (*p <* 0.0001, Student’s *t*-test) (Figure 1C). A subsequent increase in the NHEJ/no DSB/intersister HR class from 70.9 ± 1.8% in the *DmCtIP*^Δ*/+*^ heterozygote controls to 83.7 ± 1.5% in the *DmCtIP*^Δ/Δ^ mutants was observed (*p* < 0.00001, Student’s *t*-test) (Figure 1C). Additionally, there was a small decrease in SSA events from 5.8 ± 0.6% in the *DmCtIP*^Δ*/+*^ heterozygote controls to 3.7 ± 0.6% in the *DmCtIP*^Δ/Δ^ mutants (*p* < 0.05, Student’s *t*-test) (Figure 1C). 

### 3.2. DmCtIP Drives Short and Extensive End Resection in Homology-Directed SSA Repair 

**Figure 2:**
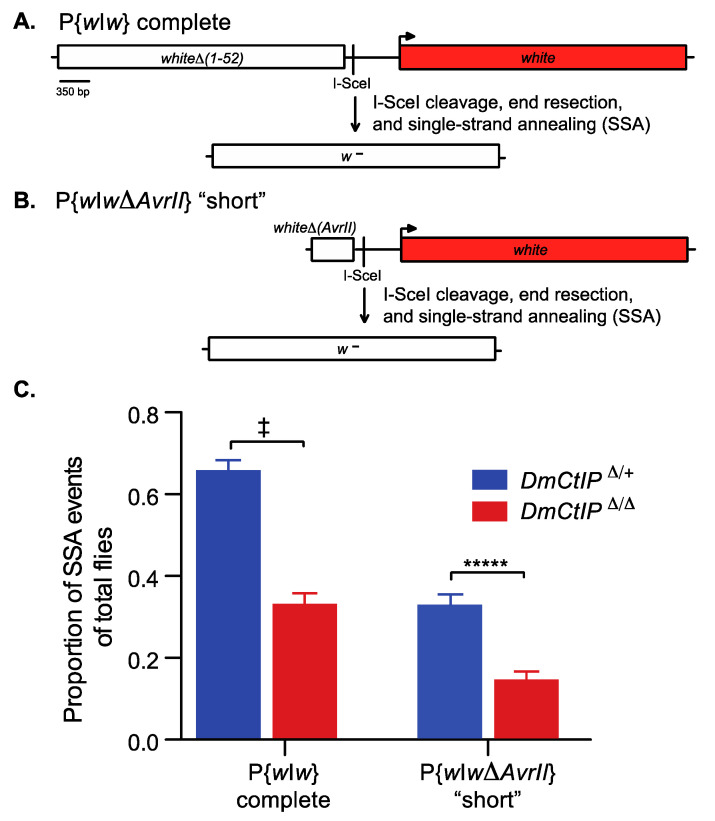
*DmCtIP*^Δ/Δ^ mutants are deficient in SSA repair in both short and long end-resection. The P{*w*I*w*} SSA assays contain an I-SceI recognition sequence inserted between a non-functional (white box) and a functional (red box) copy of the *white* gene (full-length gene sequence except for 5′UTR). (**A**) The complete P{*w*I*w*} SSA assay contains a non-functional *white* gene (*white*Δ*(1–52);* white box) due to deletions of the first 52 bp, including the ATG start codon. Following I-SceI cleavage, end resection of ~3.6 kb and single-strand annealing, a single non-functional copy of *white* results (*w^−^*) due to loss of the ATG site. (**B**) The “short” P{*w*I*w*Δ*AvrII*} SSA assay contains a non-functional *white* gene (*white*Δ *(AvrII);* white box) due to deletions of the first 52 bp (including ATG) and the last 3.0 kb (3′ end and 3′UTR). Following I-SceI cleavage, end resection of ~550 bp and single-strand annealing, a single non-functional copy of *white* results (*w^−^*) due to loss of the ATG site. (**C**) Embryos containing the respective P{*w*I*w*} constructs and a heat-shock inducible *I-SceI* transgene are heat shocked, creating a site-specific DSB, and males are crossed to *y w* tester females to score individual premeiotic germline repair events. I-SceI-induced DSB repair events from the complete P{*w*I*w*} assay in a *DmCtIP*^Δ/Δ^ mutant background (red; *n* = 23 germlines, 1708 total flies scored) compared to *DmCtIP*^Δ*/+*^ heterozygote controls (blue; *n* = 25 germlines, 1852 total flies scored). DSB repair events from the P{*w*I*w*Δ*AvrII*} assay in a *DmCtIP*^Δ/Δ^ mutant background (red; *n* = 22 germlines, 1361 total flies scored) compared to *DmCtIP*^Δ*/+*^ heterozygote controls (blue; *n* = 26 germlines, 1957 total flies scored). Results shown are averages ±S.E.M. of individual male germline events. ^‡^ *p* < 10^−10^; ***** *p* < 0.00001 by unpaired Student’s *t*-test.

End resection is required to initiate efficient homology-directed repair in HR and SSA [40]. To elucidate the impact of DmCtIP on end resection in homology-directed SSA repair, we used the *P*{*w*I*w*} homology-directed SSA assay [32]. Since end resection in homology-directed repair is a two-step process that includes both initial and extensive end resection, we employed two versions of the *P*{*w*I*w*} assay to distinguish how DmCtIP functions under short and extensive end resection requirements [18,19]. Briefly, this assay includes a *P* element with two tandem *white* sequences and an intervening I-SceI recognition site to induce DSBs by expression of a heat shock-inducible *I-SceI* transgene (Figure 2A). In the extensive end resection version of this assay, the upstream copy is non-functional due to deletion of the promoter and the first exon, while the downstream *white* gene is functional. For a homology-directed SSA event to occur in the complete version, ~3.6 kb of end resection is required. This end resection reveals sequence complementarity, and the DNA strands can be annealed through SSA, resulting in the loss of function of the downstream *white* gene and white-eyed (*w^−^*) progeny (Figure 2A). Alternatively, the P{*w*I*w*Δ*AvrII*} version of the assay contains only intron 1 of the *white* gene upstream (Figure 2B). As such, ~550 bp of end resection is required for a homology-directed SSA repair event. The resulting progeny are white-eyed (*w^−^*), losing the functional downstream *white* sequence. Red-eyed (*w^+^*) progeny, in both the complete P{*w*I*w*} and the “short” P{*w*I*w*Δ*AvrII*} versions, suggest no SSA or no DSB. Furthermore, red-eyed (*w^+^*) progeny can occur by end joining with little or no resection. Likewise, white-eyed (*w^−^*) progeny may result from end joining with deletion into the promoter of the downstream *white* sequence. 

We tested *DmCtIP*^Δ/Δ^ mutants in the complete P{*w*I*w*} and the “short” P{*w*I*w*Δ*AvrII*} assays. In the complete version (~3.6kb; extensive end resection), we found a two-fold decrease in homology-directed SSA repair, from 65.7 ± 2.5% in the *DmCtIP*^Δ*/+*^ heterozygote controls to 33.1 ± 2.6% in the *DmCtIP*^Δ/Δ^ mutants (*p* < 10^−11^, Student’s *t*-test) (Figure 2C). In the P{*w*I*w*Δ*AvrII*} version (~550 bp; minimal end resection), we expected decreased rates of homology-directed SSA repair overall in the heterozygote controls due to the reduced length of homology leading to less annealing [41]. As such, overall homology-directed SSA repair decreased in our *DmCtIP*^Δ*/+*^ heterozygote controls compared to the extensive version described above (65.7 ± 2.5% versus 32.7 ± 2.7%, respectively). However, despite the overall decrease in frequency of SSA repair in heterozygote controls, *DmCtIP*^Δ/Δ^ mutants still exhibited a two-fold decrease in SSA, from 32.7 ± 2.7% to 14.4 ± 2.1% in the *DmCtIP*^Δ/Δ^ mutants (*p* < 10^−5^, Student’s *t*-test) (Figure 2C). 

### 3.3. DmCtIP Does Not Determine Gene Conversion Tract Length in Noncrossover HR

**Figure 3:**
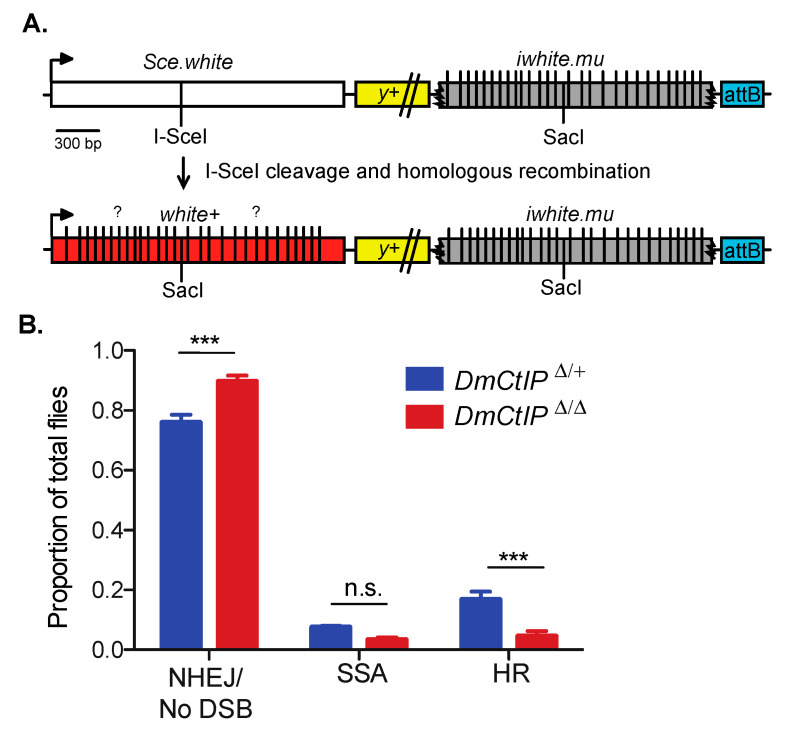
DR-*white.mu* assay shows defect in homologous recombination in *DmCtIP^Δ/Δ^* mutants. **(A**) The direct repeat of *white with mutations* (DR-*white.mu*) assay contains 28 silent polymorphisms (vertical lines) on the *iwhite.mu* donor sequence. In intrachromosomal HR events, the polymorphisms converted from the *iwhite.mu* donor sequence vary (question marks) and can be determined through PCR amplification and sequencing to determine minimal gene conversion tract (GCT) lengths. (**B**) I-SceI-induced DSB repair events in *DmCtIP*^Δ/Δ^ mutant background (red; *n* = 14 germlines, total of 631 flies scored) compared to *DmCtIP*^Δ*/+*^ heterozygote controls (blue; *n* = 26 germlines, total of 1487 flies scored). Results shown are means ± S.E.M. of individual male germline events. *** *p* < 0.001 by unpaired Student’s *t*-test.

Gene conversions that result from noncrossover HR can have significant impacts on genome stability and may result in loss of heterozygosity, with large implications for evolution and cancer [42,43]. To discern whether DmCtIP impacts the length of GCTs, we utilized the DR-*white.mu* assay [26]. This assay largely operates in the same manner as the DR-*white* assay, although the *iwhite.mu* donor sequence contains 28 silent polymorphisms that allow us to estimate minimal gene conversion tract length by PCR amplifying and sequencing across the converted SacI site of noncrossover HR repair events (Figure 3A). 

**Figure 4:**
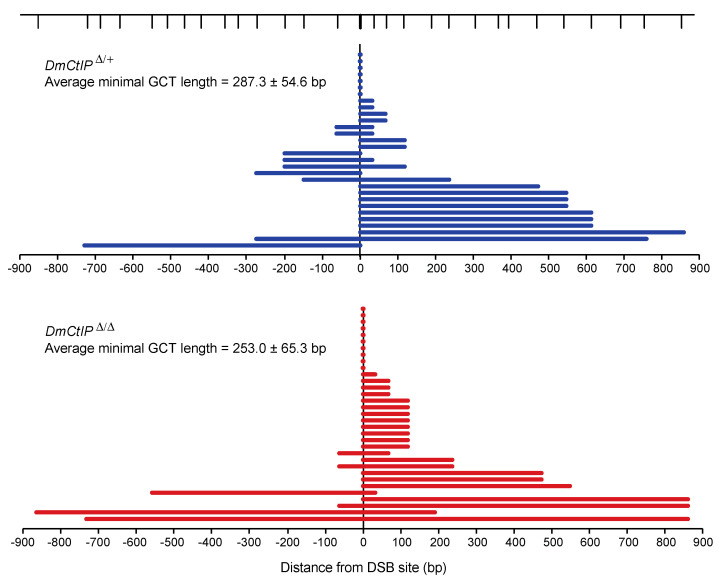
DmCtIP does not impact gene conversion tract (GCT) lengths. HR repair events result in GCTs in both *DmCtIP*^Δ/Δ^ mutants (red, *n* = 33) and *DmCtIP*^Δ*/+*^ heterozygous controls (blue, *n* = 30). SNPs along the length of the donor sequence are indicated at top (vertical lines). The zero mark represents the DSB site. The last converted SNP is shown and plotted as minimal gene conversion tracts lengths. Average minimal GCT lengths ± S.E.M. are provided for each genotype (*p* = 0.69, Student’s *t*-test).

As expected, due to the presence of mismatches in the *iwhite.mu* donor sequence [26], overall HR in heterozygote controls using DR-*white.mu* decreased relative to HR frequencies observed using the DR-*white* assay (Figure 3B). Additionally, we confirmed the same trends observed in the DR-*white* assay for the *DmCtIP*^Δ/Δ^ mutants. Specifically, we found a ~75% decrease in HR events from 16.7 ± 2.7% in the heterozygote controls to 4.5 ± 1.7% in the *DmCtIP*^Δ/Δ^ mutants (*p* < 0.001, Student’s *t*-test). Likewise, SSA events decreased from 7.4 ± 0.5% in the heterozygote controls to 3.3 ± 0.9% in the *DmCtIP*^Δ/Δ^ mutants, although this trend was not statistically significant (*p* = 0.14). For the NHEJ/no DSB/intersister HR class, we saw an increase from 75.8 ± 2.7% in the heterozygote controls to 89.7 ± 1.9% in the *DmCtIP*^Δ/Δ^ mutants (*p* < 0.001, Student’s *t*-test) (Figure 3B). Interestingly, despite the decrease in HR in *DmCtIP*^Δ/Δ^ mutants, gene conversion tract analysis revealed no significant changes in average GCT length between the heterozygous controls (287.3 ± 54.6 bp) and the *DmCtIP*^Δ/Δ^ mutants (253.0 ± 65.3 bp; *p* = 0.69, Student’s *t*-test) (Figure 4). 

## 4. Discussion

### 4.1. DmCtIP Impacts DSB Repair Pathway Distribution by its Involvement in Homologous Recombination 

Mammalian CtIP initiates end resection in conjunction with the MRN complex for repair by HR, SSA, and MMEJ [10,44]. Sartori et al. (2007) previously reported depletion of CtIP led to decreased HR frequencies in human cells, most likely from the loss of the highly conserved and functionally critical C-terminal region of CtIP, which promotes the generation of ssDNA [45]. In this study, we employed the DR-*white* assay to support *Drosophila* CtIP as a protein involved in efficient HR repair, most likely through a conserved end resection function. Additionally, the decreases we observe in SSA in the DR-*white* assay further establish *DmCtIP* as integral for homology-directed repair pathways (HR and SSA). Thus, DSBs that cannot repair via HR or SSA in *DmCtIP^Δ/Δ^* mutants are alternatively repaired by NHEJ, resulting in an increase in white-eyed (*y*^+^ *w^−^*) repair events in the DR-*white* assay.

The statistically significant increase in the white-eyed (*y*^+^ *w^−^*) repair events in *DmCtIP*^Δ/Δ^ mutants may also be explained through a direct role of DmCtIP in promoting HR by actively repressing NHEJ; this would also result in the observed shift from noncrossover HR and SSA to NHEJ in *DmCtIP*^Δ/Δ^ mutants. In *Saccharomyces cerevisiae,* the CtIP ortholog, Sae2p, has been demonstrated to promote the endonuclease activity of Mre11p to remove Ku proteins, which are NHEJ promoting factors, from the DSB sites [46,47]. If this function is conserved in *Drosophila*, the failure to efficiently remove the heterodimeric Ku protein to allow for end resection in *DmCtIP*^Δ/Δ^ mutants may explain a shift from noncrossover HR to NHEJ. Moreover, the phosphorylation of human CtIP is required for its function as a co-factor to promote end resection by the MRN complex and DNA2 [17,22]. DmCtIP contains several serine and threonine residues that could be phosphorylated. If phosphorylated DmCtIP is necessary for both end resection and removal of DmKu, phospho-dead mutants may fail to both suppress NHEJ and promote HR through its end resection activity.

Related to the role of DmCtIP in directly regulating repair pathway choice, it’s unclear if the cell-cycle-dependent regulatory circuit with 53BP1-RIF1 and BRCA1-CtIP is conserved in *Drosophila.* With the absence of a *BRCA1* ortholog in *Drosophila*, it is possible that DmCtIP plays a role in removing DmRif1 from the site of the break to allow for end resection [48]. As such, a shift from noncrossover HR to NHEJ in *DmCtIP*^Δ/Δ^ mutants may be explained through the continuous promotion of NHEJ by DmRif1.

Another interpretation of our findings is that the increase in *y*^+^ *w^−^* repair events is not due to a shift from HR to NHEJ, or a failure to suppress NHEJ per se, but rather due to increased cell death of failed intrachromosomal HR events in the germline. This lack of viability would result in a loss of total attempted HR events and a proportional increase in NHEJ. However, *DmCtIP* mutants did not display a noticeable decrease in fertility when crossed out to tester females, thus we did not detect significant germline cell loss. It is possible that the shift from red-eyed (*y^+^ w^+^*) events to white-eyed (*y^+^ w^−^*) events reflects a shift from intrachromosomal HR to intersister HR using the sister *Sce.white* sequence as a donor sequence. While these intersister HR events are molecularly and phenotypically indistinguishable from an unbroken *Sce.white* sequence, there is no evidence to suggest that end resection would promote intersister HR more than intrachromosomal HR. Lastly, it is possible that DSB induction differed between the homozygous mutants and heterozygous controls. However, this experimental variability was limited by performing our heat shock induction process of both genotypes side by side. Overall, these results support DmCtIP as a required factor for efficient HR—either directly through its end resection activity required for HR or in a role to actively suppress NHEJ activity. 

### 4.2. DmCtIP Is Required for Homology-Directed SSA Repair Due to Its Role in End Resection 

To determine whether DmCtIP has a direct role in end resection, we employed the P{*w*I*w*} assay to measure SSA. SSA is a practical measure of end resection due to its mechanistically simple and RAD51-independent nature [49]. Previous studies have described DNA end resection, primarily in mammalian cells and in vitro biochemical assays, as a two-step process with an initial, limited resection of 200–300 nucleotides, followed by a more processive and extensive resection by either the EXO1 nuclease or DNA2 in conjunction with the BLM or WRN helicases [19,50]. In humans, CtIP and the MRN complex catalyze the initial resection and promote the DNA2-dependent extensive resection pathway, as CtIP promotes DNA unwinding by BLM and the motor activity of DNA2 [19]. Using the *P*{*w*I*w*} assay to assess short versus extensive end resection requirements, we expected decreased frequencies of homology-directed SSA repair in our *DmCtIP* mutants due to the established role of CtIP in facilitating end resection. With a two-fold decrease in homology-directed SSA in both versions of the *P*{*w*I*w*} assay, our results support DmCtIP as equally necessary for efficient SSA under short (~550 bp) and extensive (~3.6 kb) end resection requirements. This supports the interpretation of the DR-*white* assay results that the decrease in HR is due at least in part to the end resection activity of DmCtIP. Notably, the observed decrease in both the “short” and complete *P*{*w*I*w*} assays suggests that DmCtIP may only be required for facilitating initial end resection. Hence, the decreases in SSA in the extensive end resection assay could be due to defects in the initial end resection step that prevent extensive end resection from occurring. If DmCtIP were required for short and extensive end resection, we would expect exacerbated defects in homology-directed SSA repair requiring extensive end resection. 

### 4.3. DmCtIP Does Not Impact GCT Length in Noncrossover HR 

Gene conversion tracts are often explained as a marker of heteroduplex DNA formation followed by mismatch repair (MMR) [51]. In current models of homologous recombination, GCT length may be dependent on the amount of single-stranded DNA generated through end resection [52]. This relationship has previously been demonstrated in *S. cerevisiae exo1* mutants, with reductions in end resection associated with shorter GCTs [52]. If the decreases in homology-directed SSA repair in *DmCtIP*^Δ/Δ^ mutants are due to reduced end resection, we would have expected shorter GCTs. However, *DmCtIP*^Δ/Δ^ mutants exhibited no significant differences in GCT length compared to heterozygote controls, suggesting that the relationship between end resection and GCT length is not as simple as it may appear. As previously shown, *S. cerevisiae yku70* mutants, which have increased end resection, and *mre11* mutants, which have decreased end resection, had GCTs similar to wild-type in chromosomal context [53,54]. If DmCtIP is more involved in the short, initial end resection (i.e., like *S. cerevisiae mre11* mutants), then this could suggest that extensive end resection is the principal contributing factor to GCT length in *D. melanogaster*. Additionally, the other proposed mechanisms that could contribute to GCT length—branch migration, repair synthesis, and MMR machinery—may be more important than end resection in determining GCT length in *Drosophila* [53,55]. Since CtIP has no proposed role in any of these other mechanisms, this could explain why we found no difference in GCT length in the *DmCtIP*^Δ/Δ^ mutants. It is important to note that the GCTs analyzed in *DmCtIP*^Δ/Δ^ mutants are a subset of repair events that can complete HR; it is possible that the reported mean GCT length in the DR-*white.mu* assay is skewed due to only measuring viable, red-eyed (*y^+^ w^+^*) progeny. Additionally, extensive gene conversion tracts beyond the *iwhite* donor sequence may alter the relative phenotype distribution by resulting in white-eyed (*y^+^ w^−^*) progeny with a converted SacI sequence at the repair site, rather than a red-eyed (*y^+^ w^+^*) HR event. 

## 5. Conclusions

In summary, this work validates the use of various genetic tools to determine how DSBs are repaired in the context of a multicellular organism. We have confirmed the capacity of the DR-*white* and P{*w*I*w*} assays to capture shifts in DSB repair pathways in mutant backgrounds accurately. Accordingly, we have proposed *D. melanogaster* CtIP as an end resection factor essential for efficient homology-directed repair. Future work is necessary to determine if DmCtIP alternatively functions as an active suppressor of NHEJ. Additionally, our results show DmCtIP is directly involved in short, initial end resection, but its involvement in extensive end resection is unclear. We suggest this lack of involvement in extensive end resection could explain the absence of change in GCT length in *DmCtIP*^Δ/Δ^ mutants.
